# Recent Progress in Rapid Sintering of Nanosilver for Electronics Applications

**DOI:** 10.3390/mi9070346

**Published:** 2018-07-10

**Authors:** Wei Liu, Rong An, Chunqing Wang, Zhen Zheng, Yanhong Tian, Ronglin Xu, Zhongtao Wang

**Affiliations:** 1 State Key Laboratory of Advanced Welding and Joining, Harbin Institute of Technology, Harbin 150001, China; w_liu@hit.edu.cn (W.L.); wangcq@hit.edu.cn (C.W.); Zhengzhen@hit.edu.cn (Z.Z.); tianyh@hit.edu.cn (Y.T.); xuronglin123@163.com (R.X.); 14b909068@hit.edu.cn (Z.W.); 2 Key Laboratory of Micro-Systems and Micro-Structures Manufacturing, Ministry of Education, Harbin Institute of Technology, Harbin 150080, China

**Keywords:** nanosilver pastes, rapid sintering, spark plasma sintering, laser sintering, electric current assisted sintering

## Abstract

Recently, nanosilver pastes have emerged as one of the most promising high temperature bonding materials for high frequency and high power applications, which provide an effective lead-free electronic packaging solution instead of high-lead and gold-based solders. Although nanosilver pastes can be sintered at lower temperature compared to bulk silver, applications of nanosilver pastes are limited by long-term sintering time (20–30 min), relative high sintering temperature (>250 °C), and applied external pressure, which may damage chips and electronic components. Therefore, low temperature rapid sintering processes that can obtain excellent nanosilver joints are anticipated. In this regard, we present a review of recent progress in the rapid sintering of nanosilver pastes. Preparation of nanosilver particles and pastes, mechanisms of nanopastes sintering, and different rapid sintering processes are discussed. Emphasis is placed on the properties of sintered joints obtained by different sintering processes such as electric current assisted sintering, spark plasma sintering, and laser sintering, etc. Although the research on rapid sintering processes for nanosilver pastes has made a great breakthrough over the past few decades, investigations on mechanisms of rapid sintering, and the performance of joints fabricated by pastes with different compositions and morphologies are still far from enough.

## 1. Introduction

Die-attach materials play a key role in ensuring the performance and reliability of electronic devices [1,2,3,4], such as in thermal [5,6,7,8] and electrical management [9,10] for high power devices. Die-attach materials are generally classified as conductive adhesives, solder alloys, glasses, metal films, and metal pastes [5]. Nowadays, conductive adhesives [11,12,13,14] and tin (Sn) based solder alloys [15,16,17,18] are most commonly used as die-attach materials for level-1 interconnections. However, these materials are only suitable for low-temperature range applications due to a low value of performance index, *M* (0.1–1.8 × 10^6^ W/m, *M* = *K*/*α*, where *K* is the thermal conductivity, and *α* is the coefficient of thermal expansion) [5], and low melting points (<250 °C) [19]. With the transition of a microelectronic system towards high power or superpower, high density integrated circuits and nano-structure interconnections, new die-attach materials and processes, which can realize low-temperature sintering and high-temperature application, should be developed [20,21]. In addition, a lead-free packaging process for microelectronic components and micro-systems is an inevitable trend in electronics industry [22,23,24,25]. Nanosilver pastes with high thermal and electrical conductivity, low sintering temperature [9,26,27,28,29,30] and high operating temperature [31] have great potential to meet the requirements of the new generation of electronics [32].

Traditional hot-pressing sintering processes for nanosilver pastes needs to apply external pressure and complicated temperature profiles, and the processes are usually time-consuming and sometimes require an inert gas atmosphere [33], which severely limit the applications of nanosilver pastes [34]. In this regard, many rapid sintering processes have been proposed to overcome the drawbacks of the hot-pressing sintering processes, such as in-situ formation of nanoparticles and joints, spark plasma sintering (SPS), laser sintering, and current assisted sintering process. Nanosilver particles can be directly interconnected by in-situ generation methods. During the process, the nanosilver particles will in-situ form at the bonding interfaces, and the particles will be relatively less affected by organic carriers. As a result, sintering temperature and time of the nanoparticles can be lowered obviously. Mu et al. obtained joints with strength of 60 MPa by using the in-situ generation method, and the bonding parameters are 5 min at 250 °C with the pressure of 5 MPa [35]. SPS is a rapid sintering technology developed in recent years. The SPS technology combines the effects of hot-pressing, resistance heating, and plasma activation. Through a SPS process, joints with shear strength of 50 MPa can be obtained when the sintering temperature is 200 °C and the sintering time is as short as 1 min [36]. Laser sintering techniques have the characters of high density of energy input and rapid heating. Sintering of nanosilver pastes can be realized in 10 s by laser irradiation, and shear strength of the sintered joints can reach 10 MPa [37,38]. Current assisted sintering technology can provide enough heat to achieve the desired sintering temperature in a short sintering time. By using electric current assisted sintering processes, interconnections can be accomplished within 1.4 s and shear strength of the joints can reach 90 MPa [34]. In this review, the mechanism of nanosilver sintering, synthesis of nanosilver, and recent progresses in rapid sintering of nanosilver pastes were discussed. Emphasis was placed on the properties of sintered joints obtained by different sintering processes.

## 2. Sintering Mechanism of Nanosilver Particles

**Figure 1:**
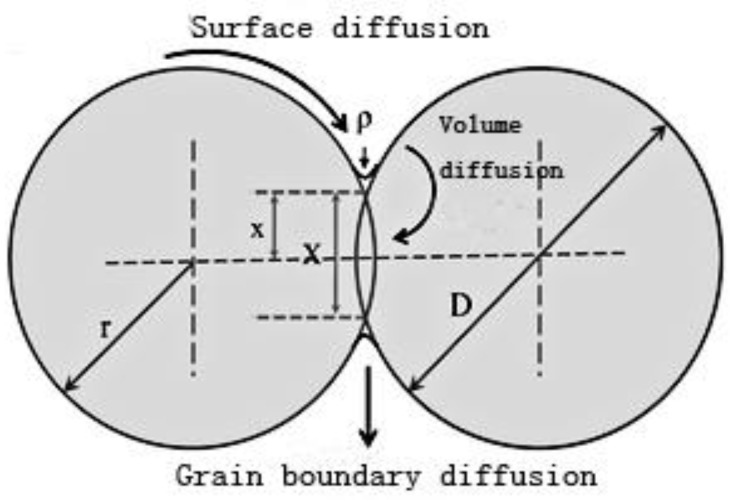
Schematic diagram of the sphere-to-sphere model. Reproduced with permission from [49].

Nanosilver particles have attracted considerable interest as one of the most promising interconnecting materials. Therefore, sintering mechanisms of nanosilver particles have become a hot topic during last few decades [20,39,40,41,42,43,44]. Various sintering models have been developed to explain the sintering mechanisms [45,46,47]. The classical sphere-to-sphere model, which has been first described by Frenkel [45], reveals that the sintering process begins with rapid neck formation, and is followed by neck growth [48,49,50]. In the initial stage, two equal-sized spheres (with radius *r*) come into contact as shown in Figure 1, to form a circular neck (with radius *x*). Subsequently, the neck begins to grow through different mechanisms of material transportation, which consists of volume diffusion, grain boundary diffusion, surface diffusion, and viscous flow during the sintering process [45,47].

The sintering equations for different sintering mechanisms can be generally expressed as follows [47]:

(1) $${\left(x/r\right)}^{n}=Bt$$

where *x*/*r* is ratio of the neck radius to the particle radius. *B* is a constant which depends on the particle size, temperature, and geometric and material terms. *t* is the sintering time and *n* is a mechanism-characteristic exponent that is depend on the mass transport process (viscous flow: *n* = 2; volume diffusion: *n* = 4–5; grain boundary diffusion: *n* = 6; surface diffusion: *n* = 7).

**Figure 2:**
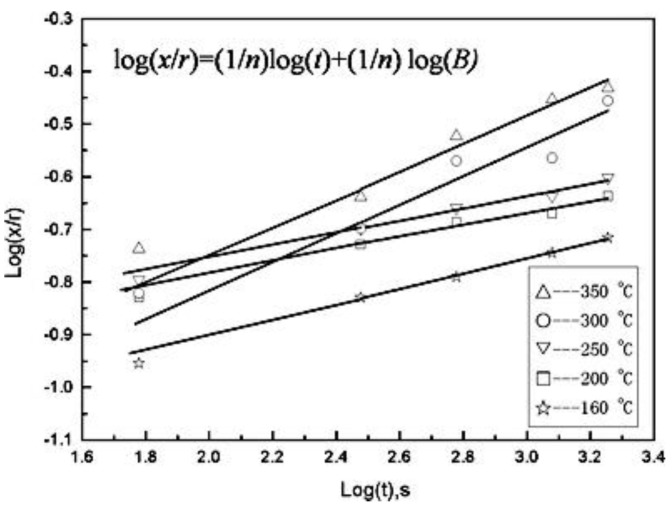
Neck growth kinetics during the sintering process of nanosilver particles at different temperatures. Reproduced with permission from [51].

In order to investigate the dominant sintering mechanism of nanosilver particles, the relationship between neck diameter and time is established. Figure 2 shows the experimental logarithm plots of the evolution of the interparticle neck size ratio *x*/*r* at different temperatures. The mechanism-characteristic exponent (the values of inverse slope) at the sintering temperatures of 160, 200, and 250 °C are 6.7, 8.8, and 8.4, respectively (the mean value is 7.9). These results indicate that surface diffusion may be the dominant diffusion mechanism at the sintering temperature range of 160–250 °C. When the sintering temperatures increase to 300–350 °C, volume diffusion is probably the prevailing diffusion mechanism [51].

Besides growth of the neck, the sintering mechanism also comprises the decomposition of the organic coating on the silver particles. Fourier-transform infrared spectroscopy (FTIR) analysis was performed to investigate the change of organic residues in nanosilver pastes during sintering processes [51]. The organic materials coated on silver particles play an important role in affecting the sintering mechanisms. By taking the Polyvinylpyrrolidone (PVP) as an example, as the sintering temperature is below 250 °C, the PVP still coats on the silver particles, and the surface diffusion is the dominant diffusion mechanism. When the temperature is increased above 300 °C, the PVP is destroyed, and the main sintering mechanism changes to volume diffusion. This indicates that the sintering mechanisms may be related with the decomposition of organic components in the nanosilver pastes. When alkylamine is utilized as a dispertant, the alkylamine will evaporate from 130 °C, thereby facilitating a low temperature sintering process of nanosilver particles [52].

Based on the classical sphere-to-sphere model, Yan et al. have revealed the relationship between the strength of joints and the neck growth of silver particles [51]. Basically, the strength of joints depends on the inherent strength of the material *τ_o_* and percent of the bonding interface, which should be proportional to the ratio of the effective bond area between the adjacent particles. The bonding area between the two contacting particles (*s*) is calculated as follows:

(2) $$s=\pi x^{2}$$

where *x* is the neck radius and the area of the sphere section of the initial particles (*S*) is given as follows:

(3) $$S=\pi r^{2}$$

where *r* is the initial radius. Therefore, the ratio of effective bond area (*R*) in each particle is expressed as follows:

(4) $$R=\frac{s}{S}=\frac{\pi x^{2}}{\pi r^{2}}={\left(\frac{x}{r}\right)}^{2}$$

According to the transverse rupture strength model [53], the joint strength is also related to the fractional density *V_s_*, the effective number of bond *N_c_/*π and the stress concentration factor *K*. Thus, it is suggested that the shear strength (*τ*) is expressed as follows:

(5) $$\tau =V_{S}(\frac{N_{C}}{K\pi })\tau_{0}R=V_{S}(\frac{N_{C}}{K\pi })\tau_{0}{\left(\frac{x}{r}\right)}^{2}$$

According to the model, the strength of the sintered nanosilver joints is proportional to the ratio of effective bond areas between the adjacent particles. The ratio of effective bond areas usually increases by elevating the sintering temperature and pressure, which will help to improve the strength of the joint. Yan et al. have performed shear tests of joints sintered at different temperatures. The results confirmed that the strength of the sintered nanosilver joints is proportional to the ratio of effective bond areas between the adjacent particles [51].

## 3. Preparation of Nanosilver Particles and Pastes

According to the reaction conditions, preparation methods of nanosilver particles can be divided into chemical reduction methods [54,55,56,57], micro emulsion methods [58,59,60], template methods [61,62,63], electrochemical methods [64,65,66,67,68], light induced or photocatalytic reduction methods [69,70,71], microwave or ultrasonic assisted methods [72,73,74,75,76,77], radiation reduction methods [78,79,80], and so on. Among them, the chemical reduction method is simple, fast, and more commonly used in preparation of nanosilver particles [81,82]. Therefore, nanosilver particles (less than 20 nm) are traditionally precipitated from the silver salt solution by chemical reduction. Briefly, reducing agents such as Ascorbic Acid [83], Monohydrate Hydrazine [84], Sodium Citrate [85], Dehydrate Sodium Citrate [21,86], Polyvinyl Pyrrolidone [87,88,89], Ethylene Diamine Tetraacetic Acid [90,91,92], Sodium Sulfite [54,93] or Sodium Borohydride [57,94] are added to the silver salt solution, for instance, Silver Nitrate [95,96], Silver Chloride [97,98] or Silver Ammonia Solution [99,100], and then the chemical reduction reaction will occur in a polar solvent such as Ethanol [101,102], Methanol [103,104] or Tetrahydrofuran [105,106]. Finally, the nanoparticles and the solution are separated by the centrifugal method [107]. Research shows that ethanol that is low-cost, environmentally friendly, and easy to volatilize is favorable to form small and uniform spherical nanosilver particles. Once high-quality nanosilver particles with uniform morphology and good dispersion are obtained, nanosilver pastes can be prepared. Generally, there are approaches to prepare the nanosilver pastes preparation. One is adding the dispersant, organic carrier, and diluent to an organic solvent, such as acetone or ethanol, and then adding commercial nanosilver particles into them. The mixture should be dispersed evenly by mechanical or ultrasonic assisted mixing. Finally, the organic solvent is evaporated by vacuum heating [41,108]. In general, the nanosilver pastes obtained by this method require higher sintering temperature, longer sintering time, and also need to apply high pressure during the sintering process. This is because the sintering of nanosilver particles depend on the thermal decomposition of organic carriers. However, the organic carriers are usually a long chain polymerization whose thermal decomposition temperature is above 250 °C [109]. Notably, Lee et al. found that besides the dispersant, negative pressure aging can also effectively solve the aggregation of nanoparticles, which will promote the sintering process of nanoparticles [110].

Another method is centrifugal separation [111,112]. First, silver nanoparticles are repeatedly washed to remove impurities. Afterwards, flocculant is added to destroy the balance of a solution, and then nanosilver particles precipitate. After centrifugation, a high concentration nanosilver pastes are obtained. The nanosilver pastes usually have lower sintering temperature as compared with the nanosilver pastes with an organic carrier.

## 4. Rapid Sintering Processes of Nanoparticles

Lu et al. [41,113,114] are pioneers who have carried out research in the area of nanoparticle sintering. They have utilized commercial silver particles with the average diameter of 30 nm to prepare nanosilver pastes and then realized the interconnection between the SiC chip at 234 °C for about 60 min with a certain applied external pressure. Shear strength of the joints was 17–40 MPa. In order to achieve good sintering properties of joints, a long sintering time, high sintering temperature, and external pressure are usually applied on the samples, which may hinder the application of the nanosilver pastes. Therefore, new processes need to be developed to shorten the sintering time, simplify the sintering process, and improve the sintering properties of the joints. Recently, extensive studies on the rapid sintering processes for nanosilver have been carried out [34,115,116,117]. Processes such as discharge plasma assisted sintering, laser sintering, and current assisted sintering cannot only enhance the efficiency of sintering, but also improve the properties of the sintered joints. The related research is shown below.

### 4.1. In-Situ Formation of Nanoparticles and Joints

**Figure 3:**
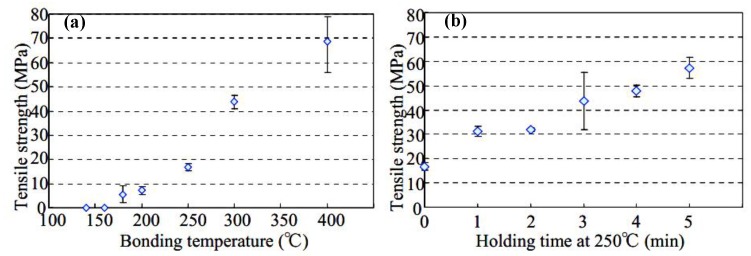
Relationship between bonding parameters and tensile strength of the joints: (**a**) Bonding temperature; (**b**) Holding time at 250 °C. Reproduced with permission from [118].

Recently, Hirose et al. and Toshiaki et al. [118,119] have proposed a novel metal-to-metal bonding process through the in-situ formation of silver nanoparticles with Ag_2_O micro-particles. During the bonding process, in-situ formation of silver nanoparticles has been achieved through a reaction between the Ag_2_O particles and triethylene glycol (TEG). The silver nanoparticles are relatively less affected by organic carriers. As a result, the sintering temperature of the nanoparticles can be lowered obviously to about 200 °C. Moreover, the cost of micron-sized Ag_2_O particles is relatively lower than commercial Ag nanoparticles. In a word, this process can both reduce cost and decrease the sintering temperature of the silver nanoparticles [35,118,120,121]. To retard migration of the Ag ion in the joints, Cu particles or Ag coated Cu particles were added into the mixed pastes [122,123,124,125]. Micron-sized Ag_2_O pastes have been successfully used in a low-temperature sintering process for the connection between silver plated copper blocks, and the sintering time can be controlled within 1 min as shown in Figure 3 [118].

### 4.2. Spark Plasma Sintering

Spark plasma sintering (SPS) is a rapid sintering technology developed in recent years [126,127,128], and the technology has many extraordinary advantages such as fast heating speed (up to 500 °C/min), and short sintering time (30–300 s) [129,130,131,132,133]. In addition, pressure is usually applied in the SPS process to help to form a better contact between nanoparticles, thereby accelerating grain boundary diffusion, lattice diffusion, and viscous flow during the sintering process [134]. All of the mechanisms could help to control the microstructure and achieve a higher density of the sintered materials. Furthermore, SPS also has the advantages of simple operation, high reproducibility, space saving, energy saving, and low cost [131,135].

**Figure 4:**
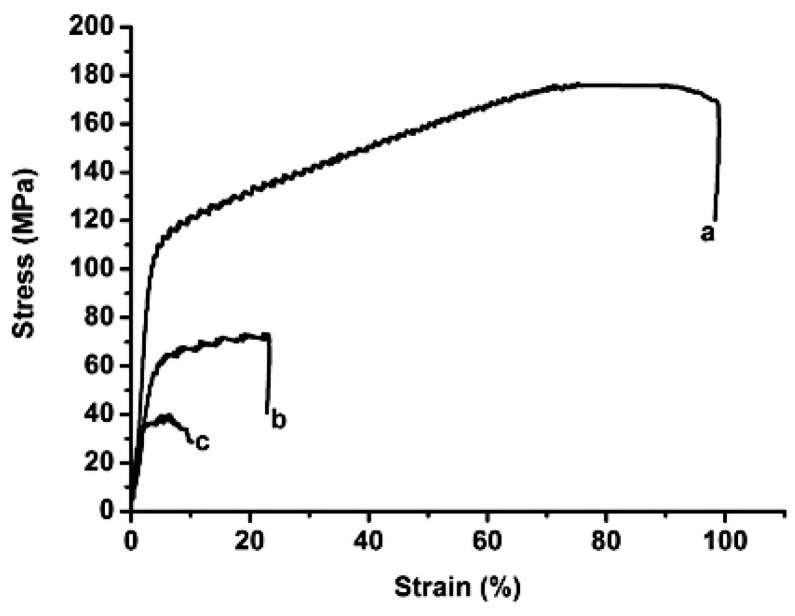
Compression tests on silver samples sintered by spark plasma sintering (SPS) at a low pressure (3 MPa), for a short dwell time (1 min), at a 300 °C, b 200 °C and c 150 °C. Reproduced with permission from [36].

**Figure 5:**
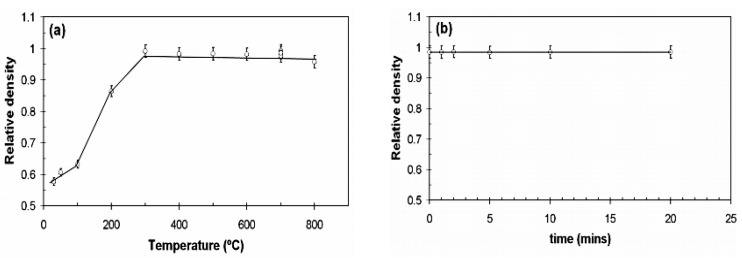
Relative density as a function of variable: (**a**) SPS temperature; (**b**) hold time. Reproduced with permission from [137].

Alayli et al. [36] used nanosilver particles and the SPS process to bond power semiconductor chips with metallized substrates. Electrical and thermal properties of the samples were both better than those sintered by conventional hot pressing processes. As shown in Figure 4, the shear strength of the joints reached 100 MPa with the sintering parameters of 300 °C, 1 min, and 3 MPa. When the sintering temperature was reduced to the range of 150–200 °C, shear strength of the joints was also as high as 30–50 MPa. Munir et al. [131] systematically summarized the influence of different parameters of SPS on properties of sintered samples. It was found that the heating rate (50–700 °C/min) had little effect on the density of the sintered samples at the same sintered temperature and time. However, the heating rate could influence the size of the sintered nanoparticles. By increasing the sintering pressure, sintering temperature could be decreased, and grain growth of the joints was also restricted. Santanach et al. [136] considered that the density of the sintered samples could be increased through prolonging the sintering time. Ng et al. [137] believed that sintering temperature could also affect density of the joints. Relative density of the samples almost reached 100% when the sintering temperature was increased to 300 °C, as shown in Figure 5.

### 4.3. Laser Sintering

Laser sintering techniques can realize fast sintering of joints with excellent properties as compared with conventional hot-pressing sintering [22,34,138,139]. At present, laser sintering techniques have been widely used in sintering processes of metal, ceramic, and composite materials [140].

**Figure 6:**
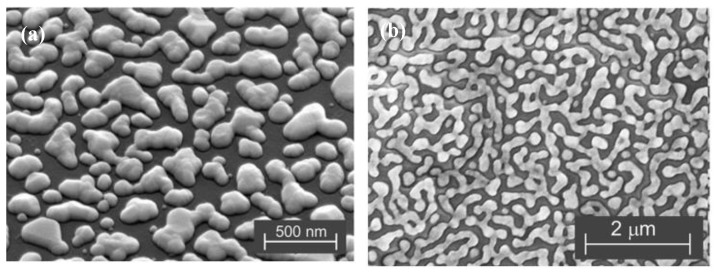
Scanning electron microscope (SEM) micrographs of nanosilver films: (**a**) heat treatment in air; (**b**) laser sintering. Reproduced with permission from [142].

Yu et al. [38] realized the bonding of a high power light-emitting diode (LED) chip (60 mil × 60 mil) with silver nanoparticles through a laser sintering process. An infrared radiation laser (30 W, *d*_spot_ = 600 μm, *λ* = 980 nm) was utilized in the study. The whole laser sintering process was 10 s after drying the organic solvent on a hot plate (230 °C, 1 min). Shear strength of the laser sintered joints could reach 9 MPa, which was higher than those fabricated by hot-pressing sintering in a convection oven (250 °C, 3 h). In addition, the LED devices showed very good performance in luminous efficiency and reliability. Liu et al. [141] have realized laser sintering dieattach processes using nanosilver pastes within 1 min. Better shear strength was obtained with increasing laser power, irradiation time, and load. Moreover, the shear strength of joints irradiated by 2–5 min of laser beam was comparable to that of the joints sintered by the hotplate for 80 min. Qin et al. [142] used a continuous wave diode pumped solid state (CWDPSS) laser to sinter thin films composed of Ag nanoparticles. The laser sintering process obtained a unique transparent conductive network structure due to the rapid heating and cooling process, whereas conventional heat treatment only formed isolated silver grains during the slow heating process, as shown in Figure 6. Liu et al. [143] successfully synthesized and transferred a transparent conductive silver film via the laser sintering process. Kunsik et al. [144] have realized laser sintering of nanosilver ink through a digital micro mirror (DMD) with high efficiency instead of the traditional printing and scanning process. Habeom Lee et al. have realized fast laser sintering of silver nanoparticle ink on plastic substrates with good properties. The laser scanning speed is 5 mm/s. In the study, the focusing lens of laser system was modified as a micro lens array or a cylindrical lens to generate multiple beamlets or an extended focal line. The modified optical settings are found to be advantageous for the creation of repetitive conducting patterns or areal sintering of the silver nanoparticle ink layer [145].

Yu et al. [146,147,148,149] compared the effects of laser type, wavelength, and power on the electrical properties and surface morphologies of sintered nano thin film. The results showed that the picosecond pulsed laser did less damage to the substrate as compared with the nanosecond pulsed laser and continuous laser. In addition, resistivity of sintered nano thin film decreased gradually, and particle size became larger with the increase of the laser power. Cheng et al. [37] simulated the ultrafast melting and re-solidification process of nanoparticles through a one-dimensional, two-temperature model. The results obtained from the model were in good agreement with the experimental data. Huang et al. [150] studied the effects of different particle size and laser frequency on the phase changes of the particles, including melting, vaporization, and re-solidification. Choi et al. [151] measured the in-situ electrical resistance of laser sintered inkjet-printed ink to study its thermal conductivity with the Wiedemann–Franz law. It was found that thermal conductivity of the sintered inkjet-printed ink would increase with the increase of laser input energy. Moreover, the thermal conductivity was also related with surface morphologies of the aggregated nanoparticles.

Up to date, different types of laser have been used in the sintering process of silver nanoparticles. However, the mechanism of laser sintering still requires further study.

### 4.4. Current Assisted Sintering

**Figure 7:**
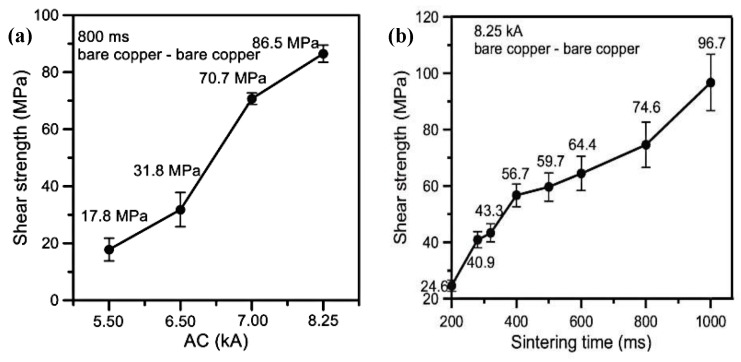
Comparison of shear strength: (**a**) Alternating Current (AC); (**b**) sintering time. Reproduced with permission from [161].

**Figure 8:**
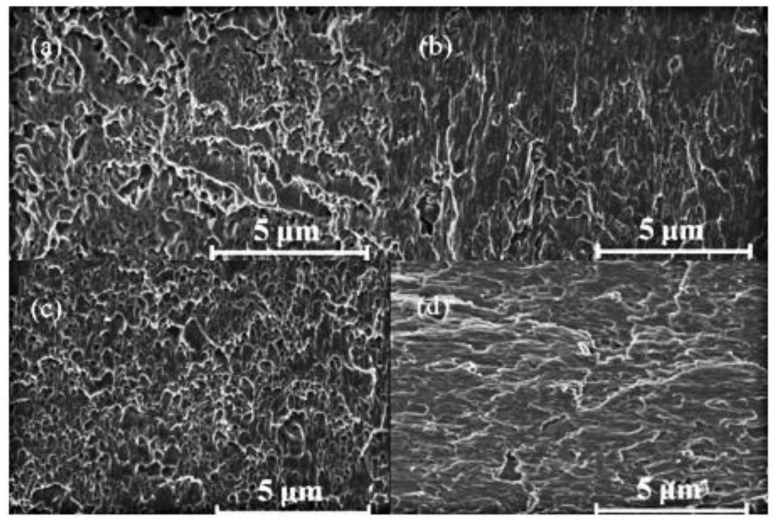
Fracture surfaces of samples sintered with different current: (**a**) 5.50 kA; (**b**) 6.50 kA; (**c**) 7.00 kA; (**d**) 8.25 kA. Reproduced with permission from [161].

Current assisted sintering technology can provide enough heat to achieve the desired sintering temperature in a short sintering time [152,153,154,155,156,157], which will restrain the coarsening of nanoparticles during the sintering process and then the fine microstructures of the joints, thereby making the joints possess good mechanical properties [158]. The shear strength of the joints fabricated by the current assisted sintering process could reach about 90 MPa with the parameters of 8.25 kA of current density for 1400 ms [159]. Mei et al. [114,160] used this technology to interconnect copper substrates with silver nanoparticles. Shear strength of the joints could reach 40 MPa within 1 s current assisted sintering. Moreover, the joints had better mechanical fatigue performance than those fabricated by traditional hot-pressing sintering methods [161]. Figure 7 shows that the shear strength of the current assisted sintered joints would increase when the current and sintering time was increased, and the maximum strength could reach 96.7 MPa. Figure 8 shows fracture surfaces of the joints. Microstructures of the joints became denser when the current was increased. Li et al. [34] found that the shear strength of the sintered joints was closely related to the peak temperature of the sintering process. Xie et al. [162] achieved robust bonding of large chips (>100 mm^2^) with nanosilver by current assisted sintering within 10 s. Moreover, thermal resistance and density of the joints could reach 0.18 °C/W and 89.6%, respectively. Transmission electron microscopy (TEM) results indicated that the better performances of the chip and joints were attributed to the high density of twins in the joints formed in the current assisted sintering process. Mei et al. [163] realized a current assisted sintering of nanosilver paste within 1200 ms, and the strength of the joints could reach 50 MPa. The current assisted sintering process could be divided into three stages: rearrangement of adjacent nanosilver particles, liquid phase assisted densification, and densification by plastic deformation and elimination of crystal defects.

Urbański et al. [164] also employed high frequency and high voltage (HFHV) electric energy to sinter nanoparticles, and applied the process to print a conductive pathway with nanosilver ink.

The reliability of sintered joints prepared by electric current assisted sintering and hot-pressing sintering were evaluated by cyclic shear test, respectively. The joints fabricated by electric current assisted sintering are more reliable than those by hot-pressing sintering [34].

## 5. Conclusions and Future Prospects

This review has summarized recent progress in the rapid sintering of nanosilver for electronics application. Emphasis is placed upon in-situ formation of nanoparticles and joints, spark plasma assisted sintering, laser sintering and electric current assisted sintering. Shear strength and microstructures of sintered joints are also discussed in terms of key process parameters, such as sintering temperature, time, current, et al. Table 1 shows the comparison of the sintering processes. The current assisted sintering could obtain relatively high shear strength in the shortest sintering time. The process of in-situ formation of nanoparticles and joints is economic because Ag_2_O is used as the raw material rather than the nanosilver. Spark plasma assisted sintering can obtain joints with high density. Laser sintering has the potential in precise selective sintering, and the process is often used to sinter nanosilver inks to form conductive networks. Current assisted sintering is usually used for connection between dissimilar materials. Moreover, the joints will have excellent shear performance and anti-fatigue properties.

Although the rapid sintering processes have many advantages as compared with conventional hot-pressing sintering processes, there are still a lot of challenges in the applications of the processes to electronic packaging. To promote the application of the rapid sintering processes, future work should focus on the following points:

(1) In some rapid sintering processes, the sintering time may be less than 1 s. The sintering mechanism of the processes may be different from that of traditional hot-pressing sintering. The sphere-to-sphere model may not be proper to explain the sintering behavior in the rapid sintering processes. Therefore, more work needs to be done to explore the mechanism of rapid sintering.
(2) In some rapid sintering processes, such as in-situ formation of nanoparticles and joints, high external pressure still needs to be applied on chips. The pressure may damage the chip during the sintering processes. Future work needs to focus on reducing the pressure applied on chips during the rapid sintering processes.
(3) Generally, binder and dispersants in the nanosilver pastes can prevent the undesirable premature coalescence or agglomeration of nanosilver particles, and the metastable structure will be retained until the organic carriers have been burned out at relatively higher temperatures. It is necessary to study the burnout characteristics of the different organics systems and design nanosilver pastes with a proper processing temperature.
(4) The bonding between different nanopaticles and metal films is a complicated process, which is related with physical, mechanical, electrostatic, diffusion, and chemical characters of the materials. Sintering parameters, such as sintering temperature, sintering time, pressure, and atmosphere will affect the bonding process and qualities of the interfaces of the materials. Future work needs to focus on the interfacial reactions and behaviors between nanoparticles and metal films during the rapid sintering processes.
(5) Currently, studies on sintering mechanisms of nanosilver particles are usually based on the spherical particle models. However, beside nanoparticles with spherical morphology, nanomaterials with other morphologies such as wires, belts, disks, and flakes are also widely mixed in pastes. The sintering mechanism of nanomaterials with different morphologies during rapid sintering processes still needs great effort.

## Figures and Tables

**Table 1:** Comparison of different rapid sintering methods.

Sintering Method	Sintering Time	Shear Strength	Cost	Ref.
Hot-pressing	30–90 min	30–84 MPa	Low	[22,165,166,167]
In-situ formation	3–5 min	50–70 MPa	Low	[35,118,120,121]
Spark Plasma	30–300 s	30–100 MPa	Medium	[36,129,130,131,132]
Laser	1–15 s	8–10 MPa	High	[38,168,169,170]
Current	0.1–1 s	40–97 MPa	Medium	[34,115,159,160,161]
